# Intrinsic thermodynamics of ethoxzolamide inhibitor binding to human carbonic anhydrase XIII

**DOI:** 10.1186/2046-1682-5-12

**Published:** 2012-06-07

**Authors:** Lina Baranauskienė, Daumantas Matulis

**Affiliations:** 1 Department of Biothermodynamics and Drug Design, Vilnius University Institute of Biotechnology, Graičiūno 8, Vilnius LT-02241, Lithuania

## Abstract

**Background:**

Human carbonic anhydrases (CAs) play crucial role in various physiological processes including carbon dioxide and hydrocarbon transport, acid homeostasis, biosynthetic reactions, and various pathological processes, especially tumor progression. Therefore, CAs are interesting targets for pharmaceutical research. The structure-activity relationships (SAR) of designed inhibitors require detailed thermodynamic and structural characterization of the binding reaction. Unfortunately, most publications list only the observed thermodynamic parameters that are significantly different from the intrinsic parameters. However, only intrinsic parameters could be used in the rational design and SAR of the novel compounds.

**Results:**

Intrinsic binding parameters for several inhibitors, including ethoxzolamide, trifluoromethanesulfonamide, and acetazolamide, binding to recombinant human CA XIII isozyme were determined. The parameters were the intrinsic Gibbs free energy, enthalpy, entropy, and the heat capacity. They were determined by titration calorimetry and thermal shift assay in a wide pH and temperature range to dissect all linked protonation reaction contributions.

**Conclusions:**

Precise determination of the inhibitor binding thermodynamics enabled correct intrinsic affinity and enthalpy ranking of the compounds and provided the means for SAR analysis of other rationally designed CA inhibitors.

## Background

Carbonic anhydrases (CAs) are zinc-containing metalloenzymes that catalyze reversible hydration of carbon dioxide to the bicarbonate ion. The enzyme has been studied since 1930s and has become a classical model protein in biochemistry [1-5]. Recently, the interest in the enzyme has risen again. It is known now that CAs are present both in prokaryotes and eukaryotes, and are encoded by five gene families: the α-CAs (predominantly in vertebrates), the β-CAs (bacteria, algae, other), γ-CAs (archaea), δ-CAs and ζ-CAs (in some marine diatoms) [6-8]. All human CAs belong to the alpha class. There are 12 catalytically active human CA isoforms: I, II, III, VII, and XIII are cytosolic, IV, IX, XII, and XIV are associated with cell membrane, VA and VB occur in mitochondria, and VI is secreted. There are 3 CA-related proteins VIII, X, and XI that are devoid of catalytic activity due to the absence of zinc. Human CAs were reviewed in detail, including their widely different catalytic and kinetic properties, response to inhibitors, expression patterns, and application for drug design [8].

Human CAs are widely distributed in many tissues and organs. The CAs play a crucial role in CO_2_ and HCO_3_^-^ transport, pH and CO_2_ homeostasis, electrolyte secretion, biosynthetic reactions, and tumor progression. Therefore the CAs became interesting targets for pharmaceutical research [9]. However, the main drawback of available compounds is insufficient selectivity towards particular CA isozyme.

Rational design of compounds with desired binding properties requires detailed investigation of the structure-activity relationships (SAR) of the newly designed compounds. Both the thermodynamics of binding and the structure of protein-ligand complex are required for detailed understanding of the reaction and search and rational design of drug-like molecules. It is important to go beyond the determination of the *K*_*i*_ and determine the full thermodynamic profile, especially the enthalpy of binding [10]. Here we describe the intrinsic thermodynamic analysis of well known CA inhibitors, ethoxzolamide (EZA), trifluoromethanesulfonamide (TFMSA), and acetazolamide (AZM) binding to human CA XIII isozyme.

Human CA XIII isozyme has been characterized [11]. The enzyme expression in human tissues showed that CA XIII is found in several organs including the thymus, kidney, submandibular gland, small intestine, and most notably in reproductive organs suggesting involvement in the fertilization process [7,11]. The CO_2_ hydration activity showed CA XIII to be a catalyst of medium efficiency [12]. Inhibition profiles demonstrated that CA XIII is similar to CA II [13]. The crystal structure of hCA XIII and its complex with acetazolamide has been solved providing crucial structural data for inhibitor SAR [12].

Manuscripts reporting novel CA inhibitors usually list only the observed thermodynamic parameters of binding or the observed inhibition constants (*K*_*i*_) [8,14-16]. Such data is useful for the design of novel inhibitors. However, it should not be used for the SAR and correlations with structures. Proper SAR requires correlation of structures only with the intrinsic binding parameters that are dissected from linked reactions occurring simultaneously with the binding reaction. Most often, such linked reactions are protonation-deprotonation reactions occurring upon ligand binding. In the case of CAs, both the inhibitor, and the CA molecule may or may not exhibit a linked protonation reaction upon inhibitor binding. Observed and intrinsic binding parameters coincide only in some rare cases when the inhibitor is deprotonated and the zinc-linked hydroxide anion is protonated. Such situation is possible only with some CA isozymes and only with the inhibitors with the *pK*_*a*_s significantly below 7. Most sulfonamide inhibitors have their *pK*_*a*_s in the range between 8 and 11. Therefore, only small fraction of the compound is in the form that binds the protein. Despite the fact that the linked protonation reactions are largely worked-out, they are rarely used in the SAR of CA inhibitors [17-19].

By ‘intrinsic’ we mean the parameters that describe actual binding reaction. In the case of sulfonamide inhibitor binding to CAs, the actual binding components are the deprotonated sulfonamide and the CA with protonated Zn-bound water molecule. Only these two forms bind each other. However, in most cases, other forms are the most abundant in solution – sulfonamide is usually protonated and the CA contains unprotonated water molecule. The concentration of the forms that actually bind is small relative to other forms and it takes energy to convert them to active forms. The observed binding energy is therefore diminished and does not resemble true (intrinsic) energetic parameters.

Determination of the intrinsic binding constant (*K*_*b*_, Gibbs free energy Δ_*b*_*G*_*intr*_), intrinsic enthalpy (Δ_*b*_*H*_*intr*_), intrinsic entropy (Δ_*b*_*S*_*intr*_), and the intrinsic heat capacity of binding (Δ_*b*_*C*_*p_intr*_) requires significant effort and a number of measurements at various pHs and temperatures. Here we use isothermal titration calorimetry (ITC) and thermal shift assay (TSA, also called ThermoFluor®, differential scanning fluorimetry) to measure inhibitor binding to CAs. ITC has been routinely used to measure ligand-protein binding thermodynamics [20]. However, it is not appropriate for determining weak (millimolar) or very tight dissociation constants (subnanomolar *K*_*d*_s require displacement ITC [21]) and consumes extensive amounts of protein and time. In contrast, TSA is a rapid screening method used in pharmaceutical industry for the identification of binders and requires lower amounts of protein [22-27]. The method is based on the protein melting temperature (*T*_*m*_) shift that occurs upon ligand binding. The *T*_*m*_ is observed by following intrinsic or extrinsic fluorescence changes upon heat-induced protein unfolding. The employment of two techniques to determine binding reactions reduces the error of the measurements. Here we apply both techniques to determine the observed binding parameters and then estimate the intrinsic parameters that could be used for CA inhibitor SAR analysis.

## Results

### Ethoxzolamide and other inhibitor binding to hCA XIII by ITC

In order to draw any thermodynamic energetics-structure correlations, the experimentally determined binding parameters must be intrinsic. In other words, all binding-linked contributing reactions must be accounted for and dissected from the reaction of interest. The most common protein-ligand binding-linked reactions are protonation or deprotonation. Sulfonamide inhibitor binding reaction to carbonic anhydrase (CA) is linked to at least two reactions, the protonation of zinc-bound hydroxide anion, and deprotonation of sulfonamide inhibitor group.

**Figure 1:**
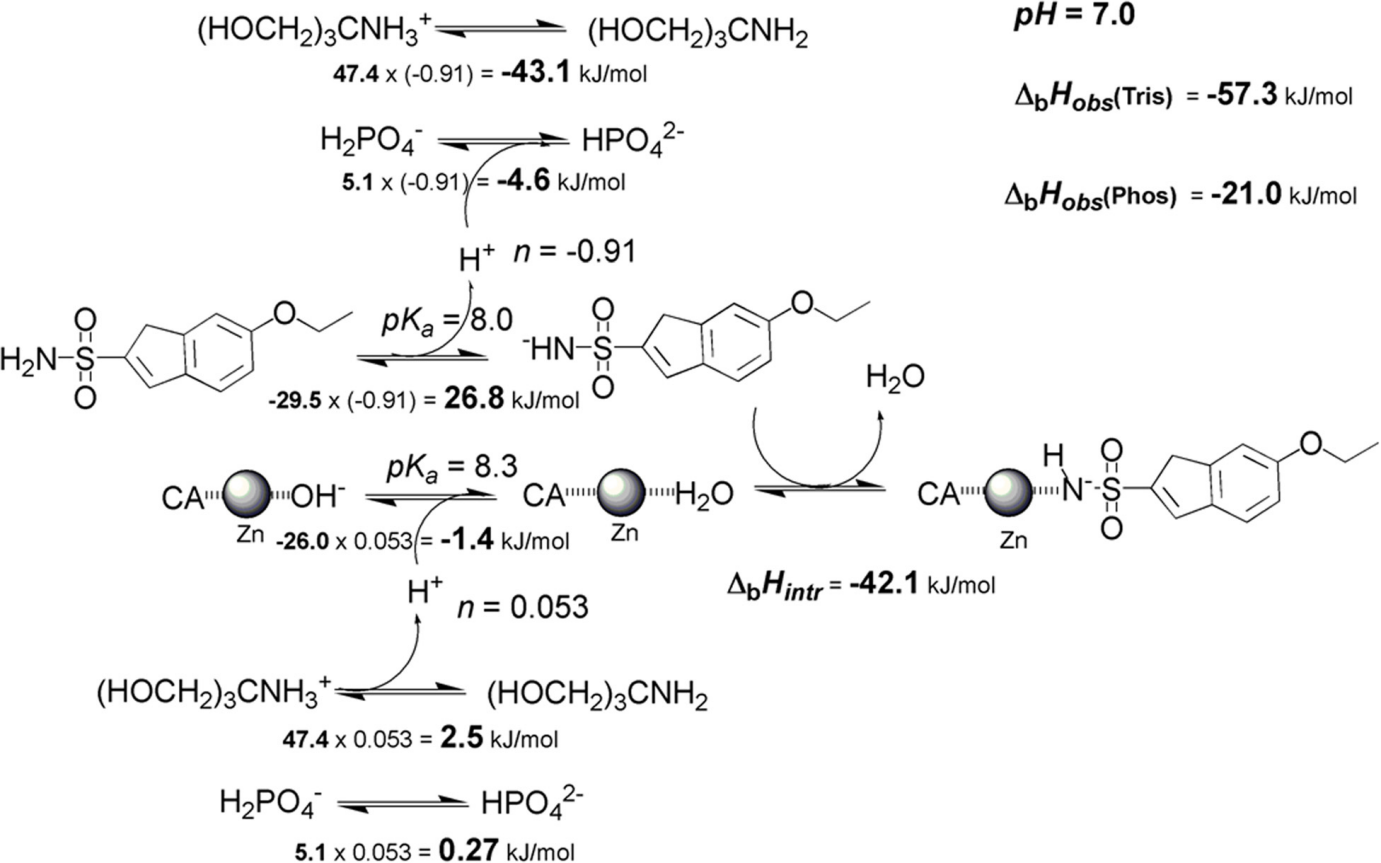
**The processes linked to the binding of EZA to hCA XIII.** Two central reactions show the deprotonation of the inhibitor sulfonamide group and protonation of the zinc-bound hydroxide to make water molecule and consecutive binding of the two active species. Two top and bottom lines show linked buffer protonation reactions. The numbers give estimates of intrinsic enthalpies for each process multiplied by the number of linked protons (*n*) yielding the observed enthalpic contribution of each reaction at pH 7.0. The numbers *n* were estimated by fitting the enthalpy dependence on pH data. The intrinsic enthalpy of binding is shown next to the reaction and is equal to −42.1 kJ/mol. The observed enthalpies are estimated for both buffers in the right upper corner. These values are similar to experimentally observed values listed in Table 1. Zinc atom is shown as grey shaded sphere and the carbonic anhydrase is shown as CA.

Figure 1 shows the reactions linked to ethoxzolamide (EZA) binding to hCA XIII. First, the sulfonamide group of ethoxzolamide must be deprotonated. Second, the zinc-bound hydroxide must be protonated. Third, the protons needed for CA and inhibitor are absorbed or donated by the buffer of the system. TRIS buffer has a large enthalpy of protonation equal to −47.4 kJ/mol while phosphate exhibits a relatively small enthalpy of approximately −5.1 kJ/mol at 25°C. Therefore, in the presence of binding-linked protonation reactions, the observed enthalpies of binding will vary significantly in these buffers and may even change sign. Obviously, protonation contributions must be dissected from binding reactions in order to obtain the intrinsic binding values.

**Figure 2:**
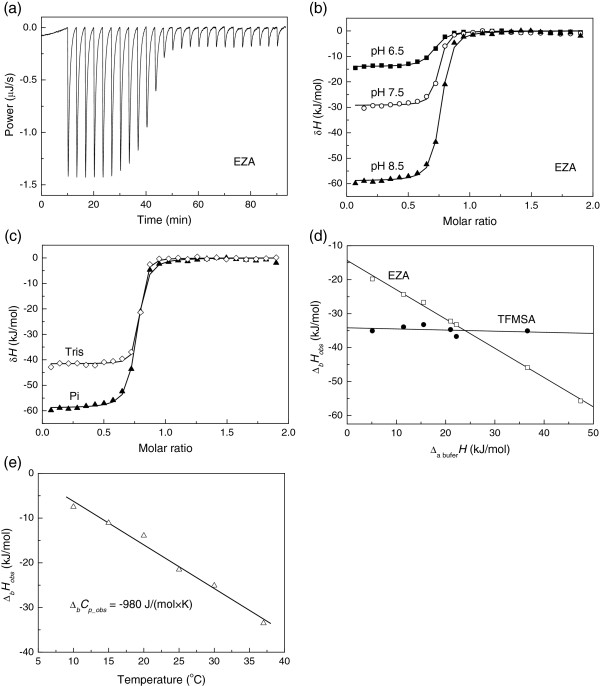
**The binding of EZA and TFMSA to hCA XIII by isothermal titration calorimetry (ITC).** (**a**) Raw ITC curve of EZA binding to hCA XIII at pH 7.0, TRIS chloride buffer, at 25°C. (**b**) Integrated ITC curves of EZA binding to hCA XIII in sodium phosphate buffer, 25°C, at several pHs: 6.5 (■), 7.5 (**◯**), and 8.5 (▲). (**c**) Integrated ITC curves of EZA binding to hCA XIII in TRIS chloride (◊) and sodium phosphate (▲) buffer, pH 8.5, at 25°C. (**d**) Observed integral enthalpies of EZA (**□**) and TFMSA (◆) binding to hCA XIII as a function of buffer deprotonation enthalpy, pH 7.0, at 25°C in the following buffers (in the order of increasing enthalpy of deprotonation, shown in the brackets, in kJ/mol): P_i_ (5.1), PIPES (11.5), MES (15.5), HEPES (21.0), MOPS (22.2), Imidazole (36.6), and TRIS (47.6). (**e**) Integral observed enthalpies of EZA binding to hCA XIII as a function of temperature. The slope is equal to the observed heat capacity of binding.

A typical isothermal titration calorimetry (ITC) raw data curve of EZA binding to hCA XIII is shown in Figure 2a. The reaction is highly exothermic and the binding is tight as seen from the steepness of the curve. The large negative peaks are due to all binding and protonation reactions while the small peaks after the middle of titration are due to dilution effects. Figure 2b shows integrated ITC curves of EZA binding to hCA XIII at various pHs in sodium phosphate buffer. The observed enthalpies of binding vary in the range of −11 kJ/mol at pH 6.0 to −66 kJ/mol at pH 9.0.

Similarly to the effect of pH, the effect of buffer is seen from Figure 2c where the observed enthalpy of binding is approximately 40% more exothermic in phosphate than in TRIS buffer. The observed enthalpies of binding vary linearly with the enthalpy of buffer protonation. Figure 2d shows EZA and trifluoromethanesulfonamide (TFMSA) binding at pH 7.0 to hCA XIII in various buffers: phosphate (5.1), PIPES (11.5), MES (15.5), HEPES (21.0), MOPS (22.2), imidazole (36.6), and TRIS (47.4) (in the order of increasing buffer protonation enthalpy in kJ/mol at 25°C in the brackets). Here, the slope of the dependence shows the net number of uptaken or released protons upon binding. The positive slope indicates that the protons are taken up from buffer upon ligand binding while the negative slope shows that the protons are given from the protein and /or ligand to the buffer. It is clear that EZA binding is linked to protonation reactions and the protons are released from the protein-ligand system to the buffer while TFMSA binding appears to be either not linked to protonation or the net number is approximately equal to zero.

Figure 2e shows the observed EZA binding enthalpy dependence on temperature at pH 7.0. The dependence is linear within the error margin of the enthalpy determination yielding the observed heat capacity of EZA binding equal to −980 J/(mol × K).

**Figure 3:**
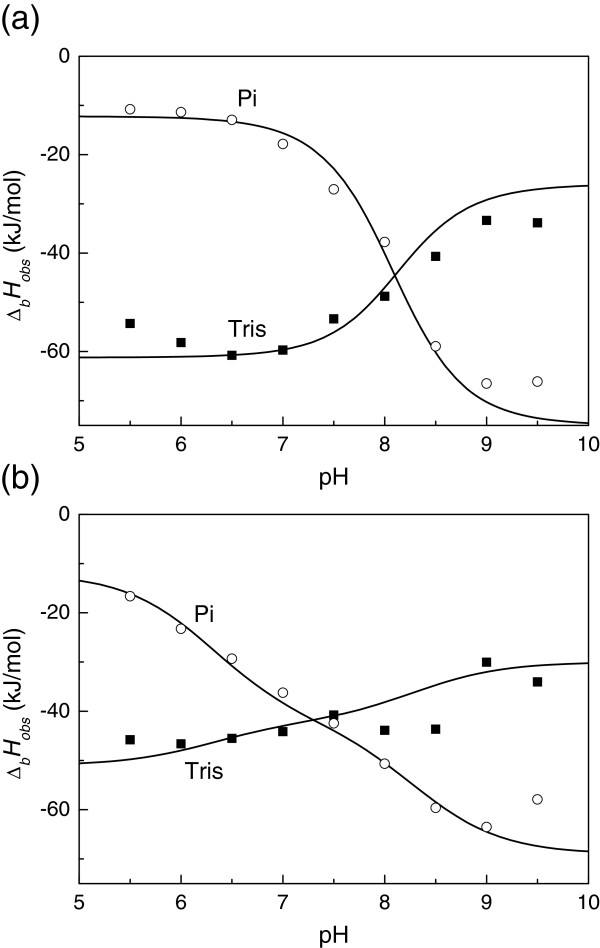
**Observed enthalpies of EZA (a) and TFMSA (b) binding to hCA XIII in TRIS chloride (**■**) and sodium phosphate (◯) buffer, 25°C, are shown as a function of pH.** Datapoints represent observed enthalpies obtained by ITC, while the curves are fitted according to Eq. (4).

Table 1 and Figure 3 show the ITC-observed thermodynamic parameters of EZA binding to hCA XIII at 25°C as a function of pH in sodium phosphate and TRIS chloride buffers. The observed parameters are significantly buffer and pH-dependent. Such parameters are experimentally observed but cannot be used for structure-activity or structure-energetics correlations. The observed enthalpies of binding vary from approximately −10 to −70 kJ/mol for EZA and TFMSA binding to hCA XIII. The values for two buffers cross each other near pH 7. Detailed dissection of linked protonation reactions is necessary to obtain the intrinsic binding thermodynamic parameters.

**Table 1:** Observed thermodynamic parameters of EZA binding to hCA XIII in phosphate and TRIS buffers are listed as a function of pH as determined by ITC at 25°C

**pH**	***K***_***b_obs***_**, M**^**-1**^	Δ_***b***_***G***_***obs***_**, kJ/mol**	Δ_***b***_***H***_***obs***_**, kJ/mol**	***T***Δ_***b***_***S***_***obs***_**, kJ/mol**	Δ_***b***_***S***_***obs***_**, kJ/(mol × K)**
EZA - hCA XIII binding in phosphate buffer					
6.0	1.70 × 10^7^	−41.27	−11.36	29.91	0.10
7.0	1.17 × 10^8^	−46.05	−17.84	28.21	0.09
8.0	4.96 × 10^8^	−49.63	−37.76	11.87	0.04
9.0	3.33 × 10^8^	−48.65	−66.48	−17.84	−0.06
EZA - hCA XIII binding in TRIS buffer					
6.0	5.95 × 10^6^	−38.67	−58.20	−19.53	−0.07
7.0	2.10 × 10^8^	−47.50	−59.71	−12.20	−0.04
8.0	8.69 × 10^8^	−51.02	−48.79	2.24	0.01
9.0	2.27 × 10^8^	−47.70	−33.36	14.33	0.05

Both the observed binding constants and enthalpies were highly pH and buffer-dependent. Standard deviations are below 10% for all parameters measured in kJ/mol.

ITC is a very good method to determine intrinsic binding parameters and dissect the linked protonation reactions. However, it must be used with great care. The main limitation is that the method has rather narrow window of experimentally determinable binding constants. If protein concentration is 10 μM, and if *c* parameter (the product of binding constant and protein concentration) is to be between 5 and 500, then the accurate observed *K*_*b_obs*_ could be obtained only from 5 × 10^5^ to 5 × 10^7^ M^-1^. As seen in Table 1, the binding is tighter at many conditions. Therefore, an additional independent method is necessary to accurately determine the intrinsic binding constant *K*_*b_intr*_, and the derived thermodynamic parameters such as the Gibbs free energy and the entropy of binding.

### EZA and TFMSA binding to hCA XIII by TSA and DSC

**Figure 4:**
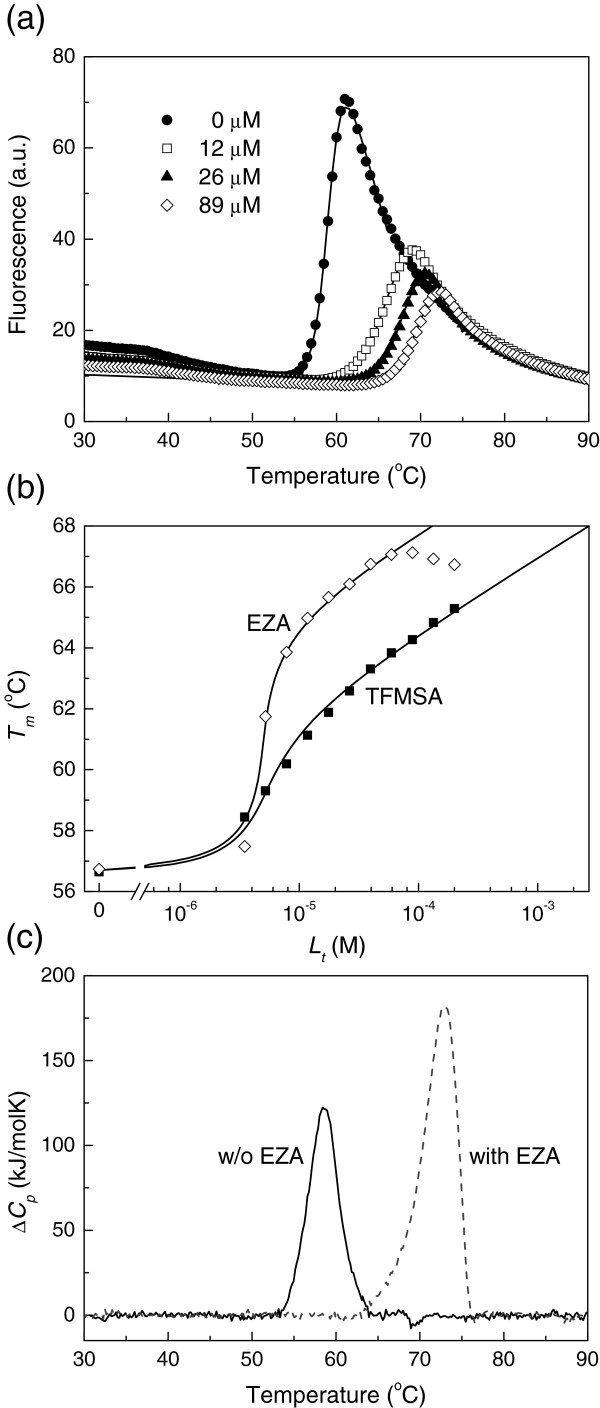
**Thermal shift assay and DSC data of EZA and TFMSA binding to hCA XIII.** (**a**) Thermal melting fluorescence curves of hCA XIII (concentration 10 μM) in sodium phosphate buffer, pH 7.0, in the presence of various EZA concentrations, 0 μM (◆), 12 μM (□), 26 μM (▲), and 89 μM (◊). Ligand addition shifted the protein melting temperatures upwards. (**b**) The dose response curves as a function of added concentration of EZA (◊) or TFMSA (■). Datapoints are obtained from fluorescence melting curves such as in panel (a), while the lines are fitted according to [27-29]. The addition of EZA shifted the *T*_*m*_s more than TFMSA at the same concentration. Therefore, the observed *K*_*b*_s are greater for EZA than for TFMSA. The possible reasons for the bending of the EZA data have been previously discussed [29]. (**c**) DSC curves of hCA XIII (50 μM) in the absence (solid line) and presence of 500 μM EZA (dashed line) in sodium phosphate buffer at pH 7.0. The addition of EZA shifted the peak to higher temperatures (thus stabilized the protein) and the area under the peak (equal to the enthalpy of unfolding) has increased due to positive heat capacity of unfolding.

A useful, simple and widely applicable method to determine ultratight binding constants is the thermal shift assay (TSA). The method measures the shift in protein melting temperature upon addition of a binding ligand. Figure 4a shows raw fluorescence curves as a function of temperature at various added EZA concentrations. The steep increase in fluorescence is due to hCA XIII unfolding and exposure of additional hydrophobic surfaces where the fluorescence of bound ANS increases. The curve midpoints represent the melting temperatures (*T*_*m*_). These midpoints shift towards higher temperatures upon addition of ligand. Addition of 100 μM EZA shifted the melting temperature by more than 10°C. The melting temperatures can be plotted as a function of temperature in a form of a ligand-dosing curve. Both the fluorescence and ligand-dosing curves can be fit as previously described [28] to accurately determine the thermodynamic parameters of unfolding and the Gibbs free energies of binding. Figure 4b compares EZA and TFMSA dosing curves. The *T*_*m*_ shift is greater for EZA than TFMSA and thus the observed *K*_*b_obs*_ is greater for EZA at the experimental conditions. The dosing curves best fitted with the enthalpy of unfolding 460 ± 100 kJ/mol (at the temperature of the *T*_*m*_ equal to 59.2°C at pH 7.0).

Similar *T*_*m*_ shift is observed by DSC (Figure 4c). Addition of EZA shifted the *T*_*m*_ by more than 10°C and the enthalpy of unfolding (the area under the DSC peak) increased due to the positive heat capacity of hCA XIII unfolding. In the absence of ligand, the hCA XIII exhibited the *T*_*m*_ equal to 58.6°C and the enthalpy of unfolding was equal to 573 ± 150 kJ/mol at pH 7.0. In the presence of 0.5 mM EZA the peak shifted to 72.4°C and the enthalpy of unfolding increased to 937 kJ/mol.

### Dissection of binding-linked protonation reactions

Here we use the same formalism to dissect protonation reactions as previously described [17-19]. The intrinsic binding constant is related to the observed binding constant and the fractions of active binding species:

(1) $$K_{b}=K_{b\_obs}/\left(f_{RSO_{2}NH^{-}}f_{CAZnH_{2}O}\right)$$

Where the fractions deprotonated sulfonamide and protonated CA as a function of pH are:

(2) $$f_{RSO_{2}NH^{-}}=\frac{10^{pH-pK_{a-\text{sulfonamide}}}}{1+10^{pH-pK_{a-\text{sulfonamide}}}}$$

(3) $$f_{CAZnH_{2}O}=1-\frac{10^{pH-pK_{a-Zn-\text{water}}}}{1+10^{pH-pK_{a-Zn-\text{water}}}}$$

The intrinsic enthalpy of binding has contributions from the observed binding enthalpy (Δ_*b_obs*_H), enthalpies of inhibitor (Δ_*b_proton_inh*_*H*), CA (Δ_*b_proton_CA*_*H*), and the buffer protonation (Δ_*b_proton_buf*_*H*):

(4) $$\Delta_{b}H_{\text{intr}}=\Delta_{b}H_{\mathrm{obs}}-n_{\mathrm{inh}}\Delta_{b\_\text{proton}\_\mathrm{inh}}H-n_{\mathrm{CA}}\Delta_{b\_\text{proton}\_CA}H+n\Delta_{b\_\text{proton}\_\text{buf}}H$$

**Figure 5:**
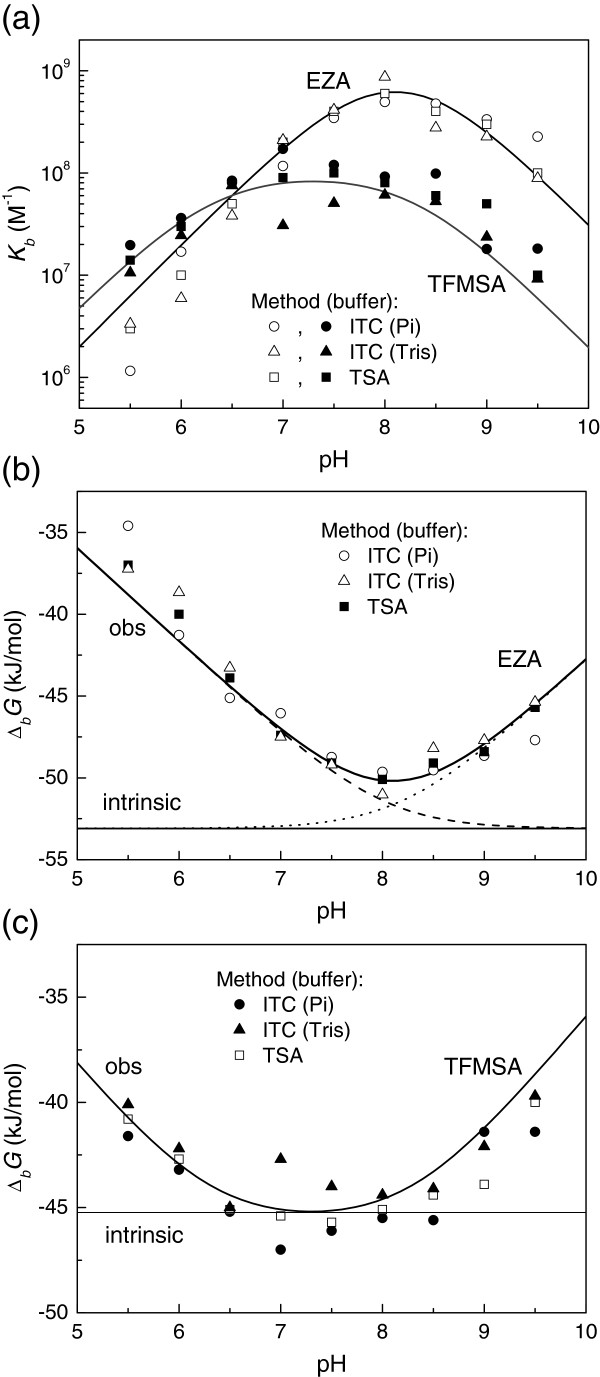
**The dependence of ligand binding constant on pH.** (**a**) Comparison of TFMSA (filled symbols) and EZA (open symbols) binding pH profiles. Curves show the fits according to Eq. (1) using parameters listed in the Tables 2 and 3. Datapoints show *K*_*b*_s obtained by ITC in phosphate (diamonds) and TRIS (triangles) buffers and by TSA (squares). (**b**) The dissection of the Gibbs free energies of EZA binding to hCA XIII as a function of pH. Datapoints are calculated from panel (a). Solid bent line shows the fit according to Eq. (1). Solid straight line shows the position of the intrinsic Gibbs free energy of binding which is independent of pH. Dashed and dotted lines show the contributions of the fractions of deprotonated EZA and protonated CA, respectively. (**c**) The observed and intrinsic Gibbs free energies of TFMSA binding to hCA XIII as a function of pH. Solid bent line is the fit according to Eq. (1) recalculated to Gibbs free energies. Straight line shows the intrinsic Gibbs free energy of TFMSA binding, independent of pH.

Figure 5a shows the EZA and TFMSA -hCA XIII observed *K*_*b_obs*_ dependence on pH. The points are experimentally (by ITC or TSA) determined while the lines are fitted according to Eq. (1). Figure 5b shows the same data for EZA plotted as Gibbs free energy. In addition, the horizontal line shows the level of intrinsic Gibbs free energy of binding and two dashed lines show contributions to the observed Gibbs free energy by the two fractions (Eqs. 2 and 3). Figure 5c compares intrinsic Gibbs free energy of TFMSA binding with the observed values. It is notable, that for TFMSA one can measure experimentally the values that are similar to intrinsic, while EZA observed values are significantly different from the intrinsic ones.

**Figure 6:**
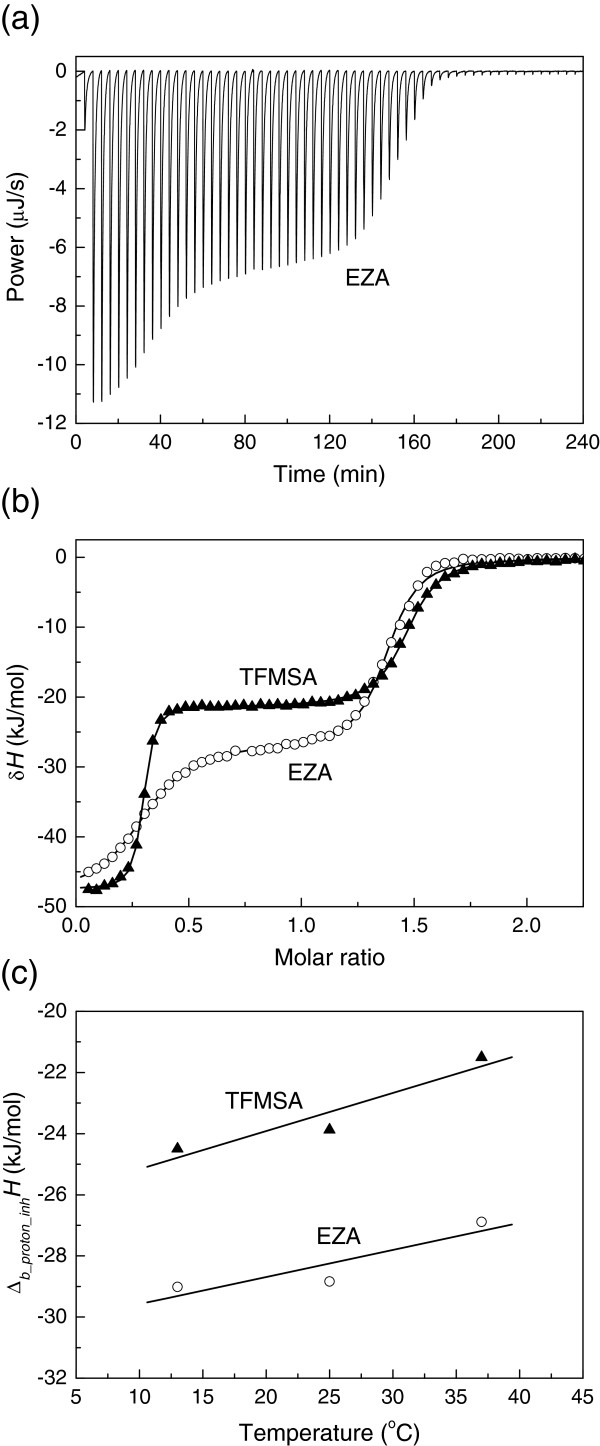
**Determination of the EZA protonation enthalpy.** (**a**) ITC raw titration curve of 0.25 mM EZA in water with 1.5 equivalent NaOH titrated with 2.5 mM HNO_3_ at 37°C. (**b**) Integrated ITC curves of EZA (**◯**) and TFMSA (▲) titration with HNO_3_ at 37°C. The first transition represents the neutralization of 0.5 equivalent of NaOH with HNO_3_ while the second transition (between the molar ratio of 0.5 and 1.5) represents the titration of the compound. In these experiments the enthalpies of protonation were −21.3 kJ/mol for TFMSA and −27.6 kcal/mol for EZA. (**c**) The enthalpies of EZA (**◯**) and TFMSA (▲) protonation as a function of temperature. The slopes yield the heat capacities of protonation equal to 88.7 J/(mol × K) for EZA and 124.7 J/(mol × K) for TFMSA.

Dissection of contribution from inhibitor protonation requires a set of experiments with pure inhibitor in the absence of protein to determine the *pK*_*a*_ and the enthalpy of inhibitor protonation. The deprotonation *pK*_*a*_ could be determined by pH titration as previously explained [30]. The enthalpy of protonation is determined by titrating alkaline solution of the inhibitor with acid. Figure 6 shows a typical ITC curve and the enthalpy of protonation dependence on temperature for both EZA and TFMSA. Pure crystalline inhibitor is in protonated electrostatically neutral form. Therefore, 1.5 equivalent of NaOH is added to the inhibitor solution to make it deprotonated. The ITC curve (Figure 6a, b) exhibits two transitions – the first transition is due to the titration of 0.5 equivalent of surplus hydroxide while the second transition is due to inhibitor protonation. This enthalpy of the second transition is the enthalpy of interest. The temperature dependence of EZA and TFMSA protonation is shown in Figure 6c. Table 2 shows the thermodynamic parameters of protonation of both inhibitors.

**Table 2:** Thermodynamic parameters of the protonation of inhibitor sulfonamide group and hCA XIII zinc-bound hydroxide anion at 25°C

	Δ_***b_proton***_***H*, kJ/mol**	**p*K***_***a***_	***K***_***a***_**, M**	Δ_***b_proton***_***G*, kJ/mol**	***T***Δ_***b_proton***_***S*, kJ/mol**	Δ_***b_proton***_***S*, J/(mol × K)**	Δ_***b_proton***_***C***_***p***_**, J/(mol × K)**
EZA	−29.5	8.0	1.0 × 10^-8^	−45.7	16.2	54.2	88.7
TFMSA	−23.9	6.25	5.6 × 10^-7^	−35.7	11.8	39.5	124.7
AZM^a^	−23.0	7.30	5.0 × 10^-8^	−41.7	18.7	62.6	ND
CA-Zn-H_2_O	−26.0	8.25	5.6 × 10^-9^	−47.1	21.1	70.7	ND

Standard deviations are below 10% for all parameters measured in kJ/mol.

a – data taken from [30].

The *pK*_*a*_ of ethoxzolamide protonation has been previously determined to be equal to 8.0 [31]. The enthalpy of ethoxzolamide deprotonation has been determined by ITC (Figure 6). The *pK*_*a*_s and enthalpies of other widely used inhibitors, including acetazolamide (AZM), methazolamide, dichlorophenamide, trifluoromethanesulfonamide (TFMSA), and p-amino benzene sulfonamide, have been previously determined [30].

### Intrinsic binding parameters

Intrinsic Gibbs free energy of EZA and TFMSA binding was determined by the global fit of data as in Figure 5 while the intrinsic enthalpy was obtained by globally fitting data in Figure 3. The intrinsic entropy of binding was obtained by subtracting Gibbs free energy from the enthalpy using standard thermodynamic equations. The intrinsic heat capacity of binding could be determined by carrying out all or part of ITC and TSA experiments at several temperatures. This was not fully accomplished in this set of experiments. However, because at pH 7.0 most of the hCA XIII zinc-bound water hydroxide is protonated, only a very small error would be obtained by disregarding the heat capacity of protonation of the zinc-bound hydroxide. The resultant intrinsic thermodynamic parameters of binding are listed in Table 3.

**Table 3:** The intrinsic thermodynamic binding parameters of deprotonated inhibitor anion to the protonated form of zinc-bound water molecule of hCA XIII at 25°C

	Δ_***b***_***H***_***intr***_**, kJ/mol**	***K***_***b_intr***_**, M**^**-1**^	Δ_***b***_***G***_***intr***_**, kJ/mol**	***T***Δ_***b***_***S***_***intr***_**, kJ/mol**	Δ_***b***_***S***_***intr***_**, J/(mol × K)**	Δ_***b***_***C***_***p_intr***_**, J/(mol × K)**
EZA	−42.1	1.8 × 10^9^	−52.8	10.7	35.9	−750
TFMSA	−33.5	4.6 × 10^7^	−43.8	10.2	34.3	ND
AZM	−50.4	1.9 × 10^7^	−41.5	−8.9	−29.8	ND

The intrinsic binding constants were greater than the observed ones and were equal to 1.8 × 10^9^ M^-1^ for EZA and 4.6 × 10^7^ M^-1^ for TFMSA. The enthalpies of binding were respectively equal to −42.1 and −33.5 kJ/mol. The entropies of binding of both inhibitors had a relatively small but positive contribution equal to 10.7 and 10.2 kJ/mol respectively. The binding process is therefore primarily enthalpy-driven and slightly supported by favorable entropic contribution for both inhibitors.

The intrinsic heat capacity of EZA binding to hCA XIII was determined to be −750 J/(mol × K). This is significantly different from the observed heat capacity of EZA binding at pH 7.0 (Figure 2e) equal to −980 J/(mol × K). The difference between observed and intrinsic parameters is important even for the heat capacity and again illustrates the importance of determining the intrinsic parameters.

The intrinsic binding thermodynamic parameters of acetazolamide (AZM) to hCA XIII were estimated by applying the same additivity principles from the data at single pH 7.0 and single temperature. The *pK*_*a*_ and enthalpy of the protein protonation were determined using EZA and TFMSA as ligands while the *pK*_*a*_ and enthalpy of AZM protonation have been previously determined [30] (Table 2). Using these parameters in the additivity scheme yields the intrinsic parameters listed in Table 3. Interestingly, binding of AZM had the largest intrinsic exothermic enthalpy of the three inhibitors, but the Gibbs free energy was least exergonic. Therefore, the entropic contribution to the AZM binding reaction was negative.

## Discussion

Detailed analysis with numerous ITC experiments at various pHs is needed to be performed just once with one selected strongly-binding inhibitor. In this study, such inhibitor was selected to be EZA. To confirm the intrinsic binding parameters, additional ligand, TFMSA was studied and showed that the same set of parameters is suitable to describe the binding of this ligand. The binding parameters of the third ligand, AZM, were determined from experiments at single pH 7.0. All other sulfonamide inhibitors to be designed and studied in the future will only need one set of ITC and TSA experiments at a single pH. In addition, the *pK*_*a*_ and enthalpy of the sulfonamide protonation will have to be determined.

Most studies, describing novel sulfonamide inhibitors listed only the observed *K*_*d*_s and/or *K*_*i*_s. [8,14,28,32,33]. The observed parameters provide estimates of the potency of each inhibitor at particular experimental conditions. However, these parameters are not suitable for the SAR-type studies and may mislead the rational design of novel inhibitors. Quite often, the observed *K*_*d*_s may differ by 3 orders of magnitude, but the intrinsic *K*_*d*_s may be comparable. In such cases, one may erroneously assign the greater observed potency to the protein-ligand interaction surface, while the true reason is simply the difference in *pK*_*a*_ of the ligand sulfonamide group.

A good example is the comparison of intrinsic and observed *K*_*d*_s of TFMSA and methanesulfonamide interaction with CA II. Their observed *K*_*i*_s and *K*_*d*_s at pH 7.0 are 2 × 10^-9^ M and 3 × 10^-4^ M, respectively. However, their intrinsic *K*_*d*_s are 1.9 × 10^-9^ M and 2.4 × 10^-8^ M, respectively. The difference between the observed and intrinsic parameters for TFMSA is quite negligible, but for methanesulfonamide is very large. Based on the observed parameters, one could erroneously conclude that the three fluorine atoms increase the inhibition constant by 150 000 times as compared to methane group. In reality, the fluorines only increase the intrinsic binding constant by only 12 times. In other words, the fluorines bind to CA II 12 times stronger than methane hydrogens and this value could be used for the SAR of the fluorines. The remaining part is due to the difference in compound *pK*_*a*_s and the available fractions of anionic sulfonamide at pH 7.0. Therefore, the studies presenting novel inhibitors should either determine the intrinsic parameters of binding or avoid pursuing observed *K*_*d*_/*K*_*i*_-based SAR analysis [26].

Acetazolamide is one the most widely used inhibitor to compare results between various laboratories. Here we determined the observed *K*_*d*_ for AZM binding to hCA XIII at pH 7.0 by both ITC and TSA to be 113 ± 30 nM. The intrinsic *K*_*d*_ is then calculated to be 53 nM. This insignificant difference from the observed value is due to the relatively low *pK*_*a*_ of AZM (7.30). Large fraction of AZM is in its active deprotonated form at pH 7.0. Supuran with coworkers lists the observed *K*_*i*_ of AZM binding to murine isozyme mCA XIII to be equal to 17 nM [13]. The difference may be due to the different organism - human and murine isozymes.

The example of AZM shows the case when intrinsic and observed binding parameters are quite similar and may appear to be within the error region. However, most CA inhibitors have their *pK*_*a*_s in the range between 9 and 11. Such inhibitors would exhibit observed *K*_*d*_s at pH 7.0 that are 2 to 4 orders of magnitude different from the intrinsic *K*_*d*_s. Large hidden differences in their *pK*_a_s could mislead the researchers to the erroneous reasons of the binding potency.

## Conclusions

Ethoxzolamide binding to hCA XIII is linked to several protonation reactions that must be dissected in order to estimate the intrinsic Gibbs free energies, enthalpies, entropies, and heat capacities of binding. Observed binding parameters for at least one inhibitor should be determined in a wide pH range. Combination of both methods, ITC and TSA is desired when the observed *K*_*d*_s are near the limit of precise determination range of ITC. Intrinsic enthalpy of EZA binding is −42.1 kJ/mol, intrinsic Gibbs free energy −52.8, and the intrinsic entropy of binding is 35.9 J/(mol × K).

## Methods

### Materials

Ethoxzolamide (EZA) was purchased from Aldrich (Milwaukee, WI, USA), trifluoromethanesulfonamide (TFMSA) was purchased from Alfa Aesar. Human carbonic anhydrase XIII (hCA XIII) was expressed in *Escherichia coli* and purified as previously described [16]. Ethics approval: no human or animal samples or live animals were used in this research.

### Isothermal titration calorimetry (ITC)

ITC experiments were performed using a VP-ITC instrument (Microcal, Inc., GE-Healthcare, Northampton, MA, USA). For ligand-protein binding experiments 5–20 μM (usually 10) protein solution in the cell and 50–200 μM (usually 100) ligand solution in the syringe were used. A typical experiment consisted of 25–30 injections (usually 25), 10 μL each, with 3–5 min (usually 4 min) intervals between injections. Experiments were performed at different temperatures in various buffers containing 50 mM NaCl and 0.5-2% DMSO concentration. All experiments were repeated at least twice. For the determination of ligand binding at various pHs, the solution pH was carefully checked in the presence of protein and ligand, in the cell and syringe, respectively. The buffering capacity is low at some pH values (for example, phosphate at pH 9.5 or TRIS at pH 5.5) and had to be carefully adjusted with acidic or basic components of the buffer.

### Thermal Shift Assay (TSA)

TSA experiments were performed with Corbett Rotor-Gene 6000 (QIAGEN Rotor-Gene Q) instrument using the blue channel (excitation 365 ± 20, detection 460 ± 15 nm), applying the heating rate of 1°C/min. Usually, the samples contained 20 μL of 10 μM protein, 0–200 μM ligand, 50 μM 8-anilino-1-naphthalene sulfonate, 50 mM sodium phosphate at pH 7.0, 50 mM NaCl , and 2% DMSO. The pH dependence of the *K*_*b*_ was determined in the universal buffer containing 50 mM sodium phosphate, 50 mM sodium acetate, and 25 mM sodium borate. The fluorescence and ligand dosing curves were fit as previously described [27,28]. TSA experiments were repeated at least three times. The standard deviation was below 10%.

### Differential Scanning Calorimetry (DSC)

DSC experiments were performed using MC-2 Scanning Calorimeter (Microcal, Inc., GE-Healthcare, Northampton, MA, USA). The samples of 1.2 mL (cuvette volume) contained 50 μM hCA XIII, 0 or 500 μM ethoxzolamide, 50 mM sodium phosphate (pH 7.0), and 50 mM NaCl. A scan rate of 1°C /min was used.

## Abbreviations

AZM: Acetazolamide; CA: Carbonic anhydrase; ΔbCp_intr: Intrinsic heat capacity of ligand binding; Δb_protonG: Gibbs free energy of protonation; ΔbGintr: Intrinsic Gibbs free energy of binding; ΔbGobs: Gibbs free energy of binding; Δb_protonCp: Heat capacity of protonation; Δb_protonH: Enthalpy of protonation; ΔbHintr: Intrinsic enthalpy of binding; ΔbHobs: Observed enthalpy of binding; Δb_protonS: Entropy of protonation; ΔbSintr: Intrinsic entropy of binding; ΔbSobs: Observed entropy of binding; EZA: Ethoxzolamide; ITC: Isothermal titration calorimetry; Ka: Acidity constant (proton dissociation, deprotonation constant); Kb_intr: Intrinsic binding constant; Kb_obs: Observed binding constant; nH: Number of protons in a protonation/deprotonation reaction; Pi: Phosphate buffer; TFMSA: Trifluoromethanesulfonamide; TRIS: TRIS (Trizma) buffer; TSA: Thermal shift assay.

## Competing interests

The authors declare that they have no competing interests.

## Authors’ contributions

LB participated in the design of this study, performed all experiments, and participated in the analysis of the data and manuscript writing. DM designed the study and wrote the manuscript. All authors read and approved the final manuscript.
